# Loss of p73 in ependymal cells during the perinatal period leads to aqueductal stenosis

**DOI:** 10.1038/s41598-017-12105-z

**Published:** 2017-09-20

**Authors:** Masashi Fujitani, Ryohei Sato, Toshihide Yamashita

**Affiliations:** 1 Department of Molecular Neuroscience, Graduate School of Medicine, Osaka University, 2-2 Yamadaoka, Suita, Osaka, 565-0871 Japan; 2 Molecular Research Center for Children’s Mental Development, United Graduate School of Child Development, Osaka University, 2-2 Yamadaoka, Suita, Osaka, 565-0872 Japan; 3 Department of Anatomy and Neuroscience, Hyogo College of Medicine, 1-1 Mukogawa-cho, Nishinomiya, Hyogo, 663-8501 Japan; 4 World Premier International, Immunology Frontier Research Center, Osaka University, 3-1 Yamadaoka, Suita-shi, Osaka, 565-0871 Japan; 5 Graduate School of Frontier Biosciences, Osaka University, 1-3 Yamadaoka, Suita-shi, Osaka, 565-0871 Japan

## Abstract

The p53 family member p73 plays a critical role in brain development. p73 knockout mice exhibit a number of deficits in the nervous system, such as neuronal death, hydrocephalus, hippocampal dysgenesis, and pheromonal defects. Among these phenotypes, the mechanisms of hydrocephalus remain unknown. In this study, we generated a p73 knock-in (KI) mutant mouse and a conditional p73 knockout mouse. The homozygous KI mutants showed aqueductal stenosis. p73 was expressed in the ependymal cell layer and several brain areas. Unexpectedly, when p73 was disrupted during the postnatal period, animals showed aqueductal stenosis at a later stage but not hydrocephalus. An assessment of the integrity of cilia and basal body (BB) patch formation suggests that p73 is required to establish translational polarity but not to establish rotational polarity or the planar polarization of BB patches. Deletion of p73 in adult ependymal cells did not affect the maintenance of translational polarity. These results suggest that the loss of p73 during the embryonic period is critical for hydrocephalus development.

## Introduction

The cerebrospinal fluid (CSF) ventricular system is a dynamic circulatory system that plays a critical role in mammalian brain homeostasis throughout life^1^. Abnormal accumulation of CSF leads to expansion of the ventricles, resulting in hydrocephalus. Ependymal cells are multiciliated epithelial cells that line the walls of the ventricles in the brain and generate directional CSF flow by beating their motile cilia^2^. Accumulating evidence suggests that abnormal development of these motile cilia results in hydrocephalus^2–5^. Impaired ciliary motility may disrupt CSF flow in the narrowest portion, inducing aqueductal collapse and subsequent hydrocephalus^6^. However, cilia dysfunction does not always lead to obstructive hydrocephalus^4,7,8^. In addition, whether a patent aqueduct, an aqueductal stenosis and an aqueductal occlusion are sequential phenotypes that occur during the pathogenesis of hydrocephalus remains a critical question^9^.

The p53 family member p73 plays a critical role in brain development^10–14^. Previous studies have shown that p73 null mutant mice present neuronal degeneration^15,16^, hydrocephalus^17,18^, pheromonal defects^18^, and hippocampal dysgenesis^13,14^. Among these phenotypes, the mechanisms of ventricular dilation remain unknown^15,19^. Recent studies suggest that p73 regulates ependymal cell maturation and multiciliogenesis^17,19,20^. We intended to elucidate the precise molecular function of p73 in ependymal cell maturation and multiciliogenesis using spatiotemporal genetic manipulation in developing ependymal cells.

In the present study, we generated a p73 knock-in (KI) mouse and a conditional p73 knockout mouse. As previously reported^17^, the p73 homozygous mutant mouse shows loss of ventricular integrity and aberrant translational polarity in adult ependymal cells of the p73 homozygous mutant brain. Conditional deletion of p73 in adult mice had no influence on the maintenance of translational polarity. Importantly, postnatal deletion of p73 leads to aqueductal stenosis at a later stage.

## Results

### p73 KI mouse shows hydrocephalus and aqueductal stenosis

**Figure 1:**
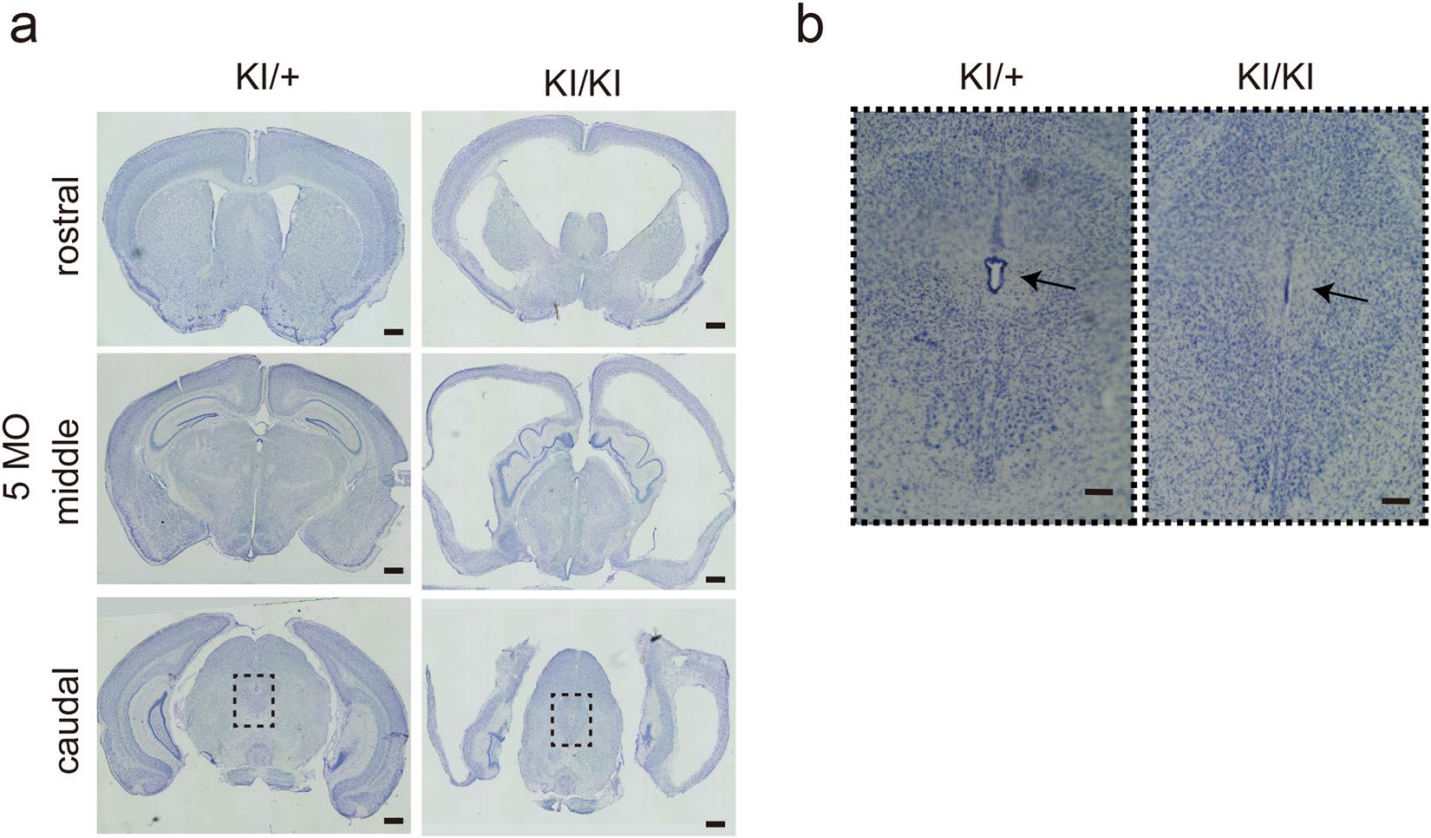
Morphological analysis of a p73 knock-in (p73 KI) mouse (**a**) Nissl staining of coronal sections of rostral, middle, and caudal parts of embryonic day (e) 18.5 brains of both heterozygous (p73 KI/+) and homozygous (p73KI/KI) mutants. Scale bars, 500 μm. (**b**) High magnification micrographs of squares illustrated in (a). Arrows indicate aqueducts in midbrain. Scale bars, 100 μm.

We first established p73 KI mice using embryonic stem cells obtained from the KOMP Repository (www.komp.org) (Supplementary Fig. 1a). By generating the KI model, we intended to determine p73 mRNA expression by X-gal staining and to generate conditional knockout animals. p73 has two promoters; one is for the full-length isoform TAp73, and another is for the N-terminal truncated isoform ΔNp73. To disrupt both isoforms, an ENGRAILED 2(EN2) splice acceptor and the Internal Ribosome Entry Site (IRES) *LacZ* cassette were inserted into intron 4 (Supplementary Fig. 1a). We confirmed complete loss of p73 mRNA expression in postnatal homozygous KI mutants, whereas 75% expression in heterozygous KI mutants were compared to wild-type mice (Supplementary Fig. 2a). We analyzed the survival curve. Consistent with observations in conventional knockout mouse^18^, approximately 90% of the homozygous KI mutants died postnatally (Supplementary Fig. 2c). We examined the brains of embryonic day (e) 18.5 homozygous KI mutants. We observed no abnormality in the cortical development of the telencephalon in heterozygous or homozygous KI mutants (Supplementary Fig. 2b), consistent with a previous finding^15,21^. However, the surviving adult homozygous KI mutants (n = 2) showed severe hydrocephalus, as previously reported^18,19^(Fig. 1a). Interestingly, we identified aqueductal stenosis in homozygous KI mutants (Fig. 1b), even though this phenotype has never been reported.

### p73 expression in adult and postnatal brains

Although p73 expression in the embryonic and postnatal mouse brain has been analyzed^18,22,23^, adult p73 expression remains to be determined. It was difficult to determine the expression of p73 in the adult nervous system due to the low level of p73 expression^24^ and the problem of antibodies that are cross-reactive to other members of the p53 family^25,26^. Thus, to resolve this problem, we determined that X-gal staining for p73 heterozygous KI mutant was highly specific and sensitive, as shown in Supplementary Fig. 3. Consistent with the previous observation^27^, this method was highly reproducible and revealed no non-specific staining in the CNS. As observed in Supplementary Fig. 3a–d, *LacZ* expression was mainly observed in the walls of the lateral ventricles (LV), the third ventricle (III), and the fourth ventricle (IV) of the brain and central canal of the spinal cord (black arrows). Higher-magnification micrographs show that p73 was also expressed in hypothalamic cells (Supplementary Fig. 3i) (black arrowhead) but not in the choroid plexus (Supplementary Fig. 3j). The sections from wild-type animals show no X-gal staining (data not shown).

On postnatal day 5 (P5), p73 expression was observed in the ventricular walls, including those for LV, III, and IV; the aqueduct (black arrows); the choroid plexus (Supplementary Fig. 3p,q); (white arrowhead); hypothalamus cells (Supplementary Fig. 3u) (black arrowhead); cortical layer I (Supplementary Fig. 3s,u); and the hippocampus (Supplementary Fig. 3q) (dotted arrows). These cortical and hippocampal cells might be Reelin-positive Cajal-Retzius cells^22^. These results prompted us to hypothesize that p73 maintains adult ependymal cells and controls multiciliogenesis during postnatal development.

### Conditional ablation of p73 in postnatal ependymal cells causes aqueductal stenosis

**Figure 2:**
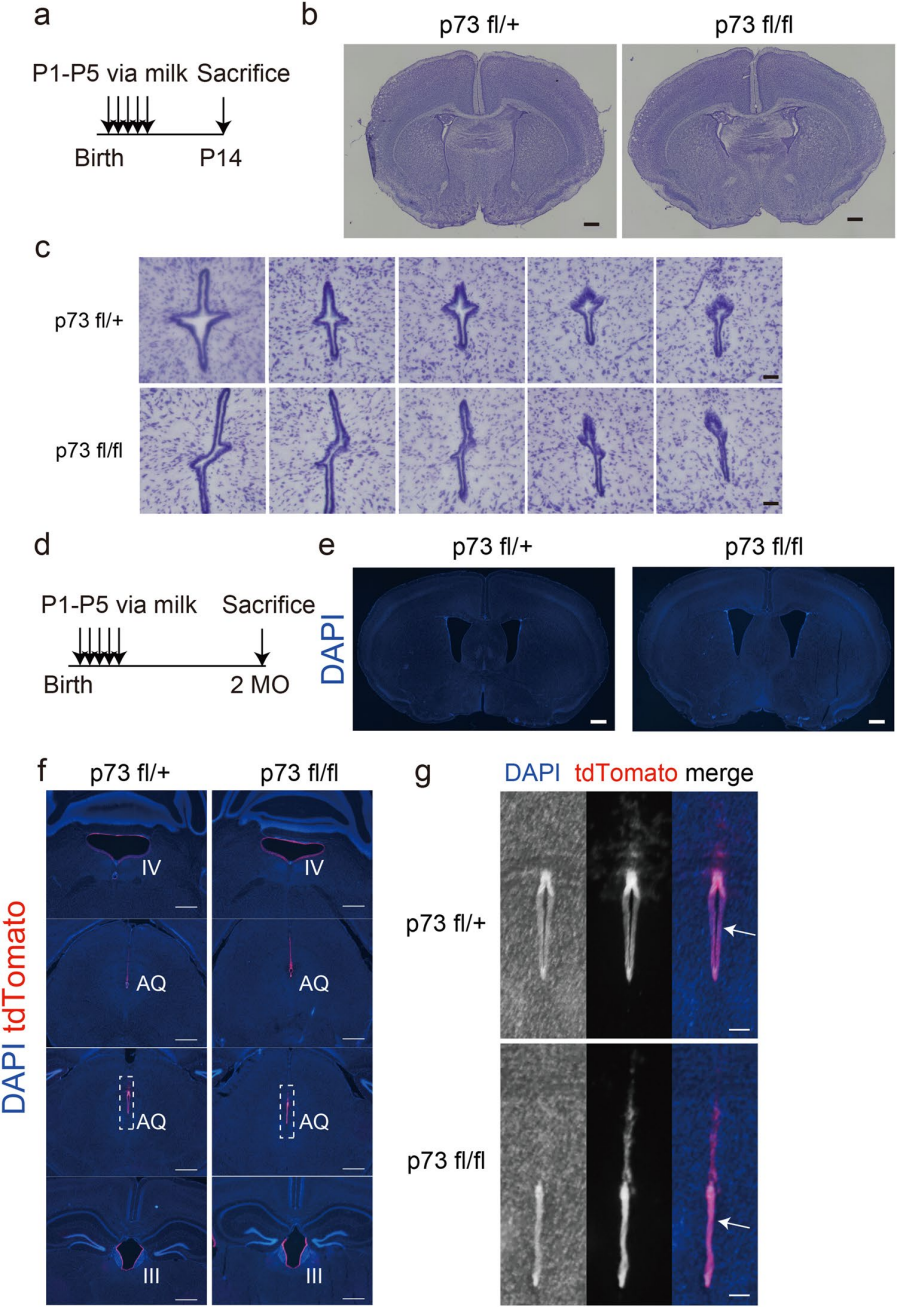
Loss of p73 in postnatal ependymal cells leads to late-onset aqueductal stenosis without hydrocephalus. (**a**) Experimental schedule used in this study. (**b**) Nissl staining of coronal sections of the rostral brain at postnatal day 14 (P14) for both heterozygous (p73 fl/+) and homozygous (p73 fl/fl) conditional mutants. Scale bars, 500 μm. (**c**) Nissl staining of coronal sections of the aqueduct (AQ) at postnatal day 14 (P14). Scale bars, 50 μm. (**d**) Experimental schedule used in this study. (**e**) DAPI staining of coronal sections of the rostral brain at 2 months old (2MO). Scale bars, 500 μm. (**f**) DAPI staining of coronal sections of the fourth ventricle (IV), AQ and third ventricle (III) of the brain at 2MO. Scale bars, 500 μm. (**g**) High magnification micrographs of squares illustrated in (f). Arrows indicate AQs in the midbrain. Scale bars, 100 μm.

To clarify p73 function in the ependymal cells of the postnatal or adult brain, we generated a p73 conditional knockout mouse (Supplementary Fig. 1b). We crossed our p73 floxed mouse with a R26R tdTomato reporter mouse, which was previously crossed with a FoxJ1Cre ERT^2^ mouse^28^. After injection of tamoxifen into this triple transgenic mouse, conditional genetic recombination occurs specifically in ependymal cells. The R26R-tdTomato reporter allele allows clear visualization of recombined cells. To confirm p73 conditional disruption, we administered tamoxifen to one-month-old mice, dissected tissues from ependymal and subventricular (E/SVZ) zones a week later, and analyzed p73 mRNA expression (Supplementary Fig. 4a). As expected, in E/SVZ samples from p73 flox homozygous mouse, we observed an approximate 90% loss of p73 mRNA compared to tamoxifen-injected heterozygous or non-injected wild-type mice (Supplementary Fig. 4a). Importantly, comparable expression of p73 mRNA in SVZ for wild-type and heterozygous animals enabled us to analyze p73 heterozygous flox (p73 fl/+) animals as a control. Motile multiciliogenesis occurs during the postnatal period in developing brains^6^. Therefore, to examine p73 function in postnatally developing ependymal cells, tamoxifen was administered to pups through the mother’s milk^29^. From postnatal day 1 (P1) to day 5 (P5), tamoxifen was injected into lactating mothers following the experimental schedules shown in Fig. 2a,d. After perfusion and sectioning of the p73 floxed heterozygous and homozygous mouse brains on postnatal day 14 (P14) and at 2 months, we performed Nissl staining (P14; Fig. 2b,c) or DAPI nuclear staining (2 months; Fig. 2e–g) after confirming tdTomato staining (data not shown). To confirm Cre recombination in FoxJ1-expressing ependymal cells, we examined tdTomato expression (Fig. 2f,g). The p73 floxed homozygous mutant brain showed no significant dilatation of the lateral ventricles and no significant aqueductal stenosis (Fig. 2b,c). We analyzed perinatal tamoxifen-administered homozygous and heterozygous mutant mouse brains at 2 months. The p73 floxed homozygous mutant brain showed no significant dilatation of the lateral ventricles at 2 months (Fig. 2e) but revealed aqueductal stenosis (Fig. 2f,g). These data suggest that loss of p73 in the postnatal ependymal cells leads to subsequent aqueductal stenosis, although apparent hydrocephalus was not observed.

### p73 is required for translational polarity formation

**Figure 3:**
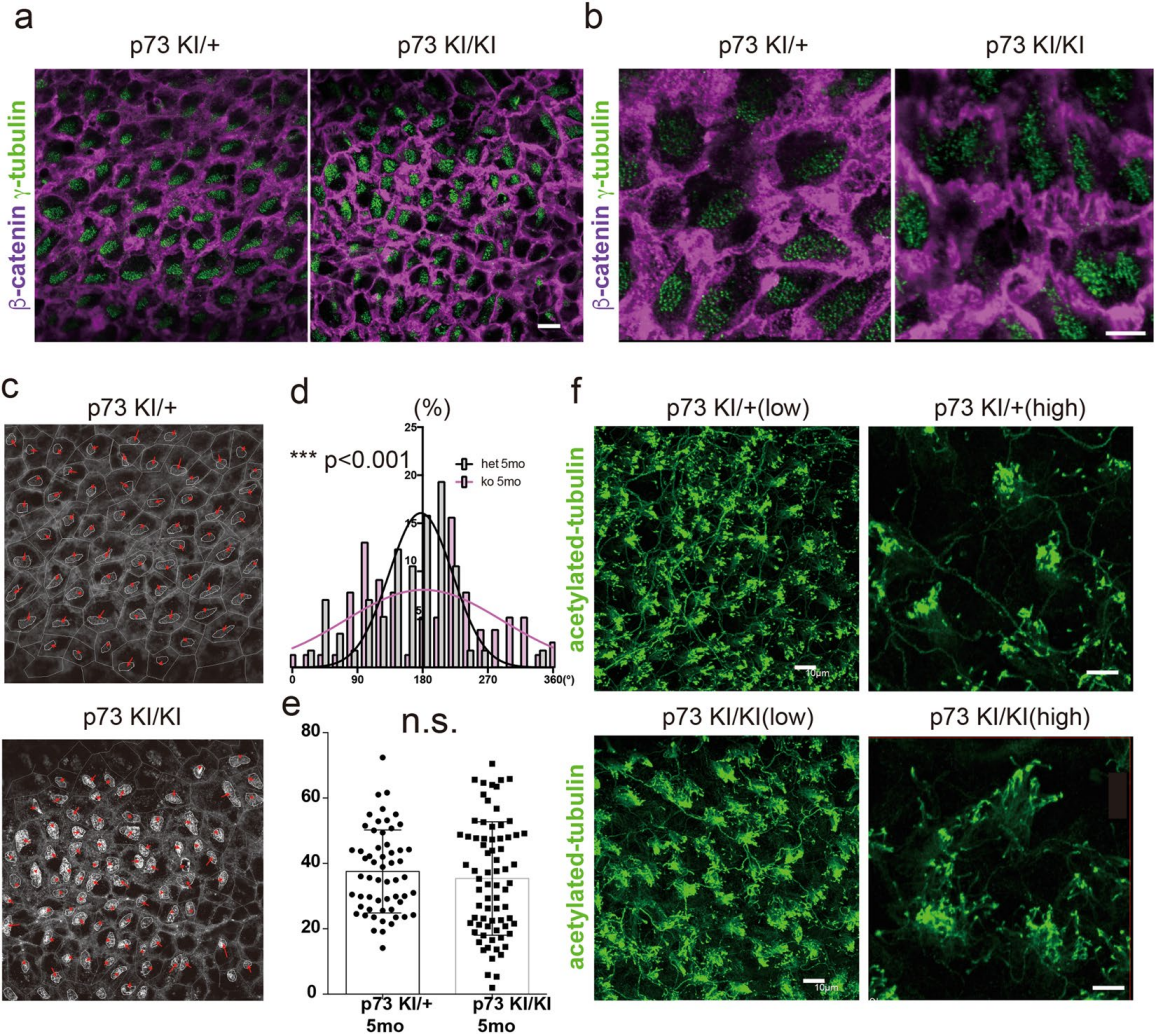
Loss of p73 leads to abnormal cytoskeletal architecture and aberrant translational polarity. (**a**) Whole-mount preparations of lateral walls of lateral ventricles stained with antibodies against β-catenin (purple) and γ-tubulin (green) in heterozygous (p73 KI/+) and homozygous (p73 KI/KI) mutant brains (scale bar, 10 μm). (**b**) Higher magnification pictures of (**a**) (scale bar, 5 μm). (**c**) Traces of intercellular junction, labeled with β-catenin, and basal body (BB), labeled with γ-tubulin, of ependymal cells are shown in (**a**). Red arrows show vectors drawn from center of the apical surface to BB patch. (**d**) Histogram of distribution of BB patch angles in heterozygous (p73 KI/+) (gray) and homozygous (p73 KI/KI) (pink) mutants. (***p < 0.001, Watson’s U^2^ test, n = 57 p73 KI/+mice, n = 73 p73 KI/KI mice). (**e**) Quantification of BB patch displacement. (n.s. not significant, mean ± SEM; n = 57 p73 KI/+mice, n = 73 p73 KI/KI mice). (**f**) Whole-mount preparations of lateral walls of lateral ventricles stained with antibodies against acetylated-tubulin (green) in heterozygous (p73 KI/+) and homozygous (p73 KI/KI) mutant brains. Left side panels show low magnification micrographs. Right side panels show high magnification micrographs. Scale bar, 10 μm (low) and 5 μm (high).

A p73 deficiency may cause abnormal differentiation of radial glia to ependymal cells and abnormal ciliogenesis, as previously suggested^17^. However, no information is available regarding the morphology of cilia in the adult p73 knockout mouse. Thus, we immunostained whole-mount preparations of the lateral walls of the lateral ventricles with antibodies against β-catenin (purple) and γ-tubulin (green) in heterozygous and homozygous KI mutant brains to examine ependymal cell and basal body (BB) morphology^30^. Ependymal cell morphology, as determined by immunostaining for β-catenin, was disrupted in the homozygous mutant mouse (Fig. 3a,b). Additionally, we immunostained γ-tubulin and analyzed BB patch displacement from the center of the apical surface using the magnitude of the BB patch vector (Fig. 3c–e)^7^. The BB patch angle, which represents translational polarity, was disorganized in p73 homozygous mutants (Fig. 3c,d), whereas the BB patch displacement ratio was not affected (Fig. 3e). These results suggest that p73 is required for establishing translational polarity, but not for the planar polarization of BB patches on the apical surface of ependymal cells.

We examined cilia morphology and axons on the surface of lateral ventricles by immunostaining with an anti-acetylated tubulin antibody (Fig. 3f). The ependymal cells displayed straight parallel bundles of cilia in both the p73 heterozygous KI mutant and the homozygous mutant (Fig. 3f). Higher magnification micrographs confirmed that the bundles of cilia in both mutants were parallel (right panels of Fig. 3f). Therefore, p73 deficiency may delay cilia formation, and translational polarity was specifically disrupted in the ependymal cells of the homozygous KI mutants.

### Conditional ablation of p73 in adult ependymal cells did not cause hydrocephalus or cilia deficiency

**Figure 4:**
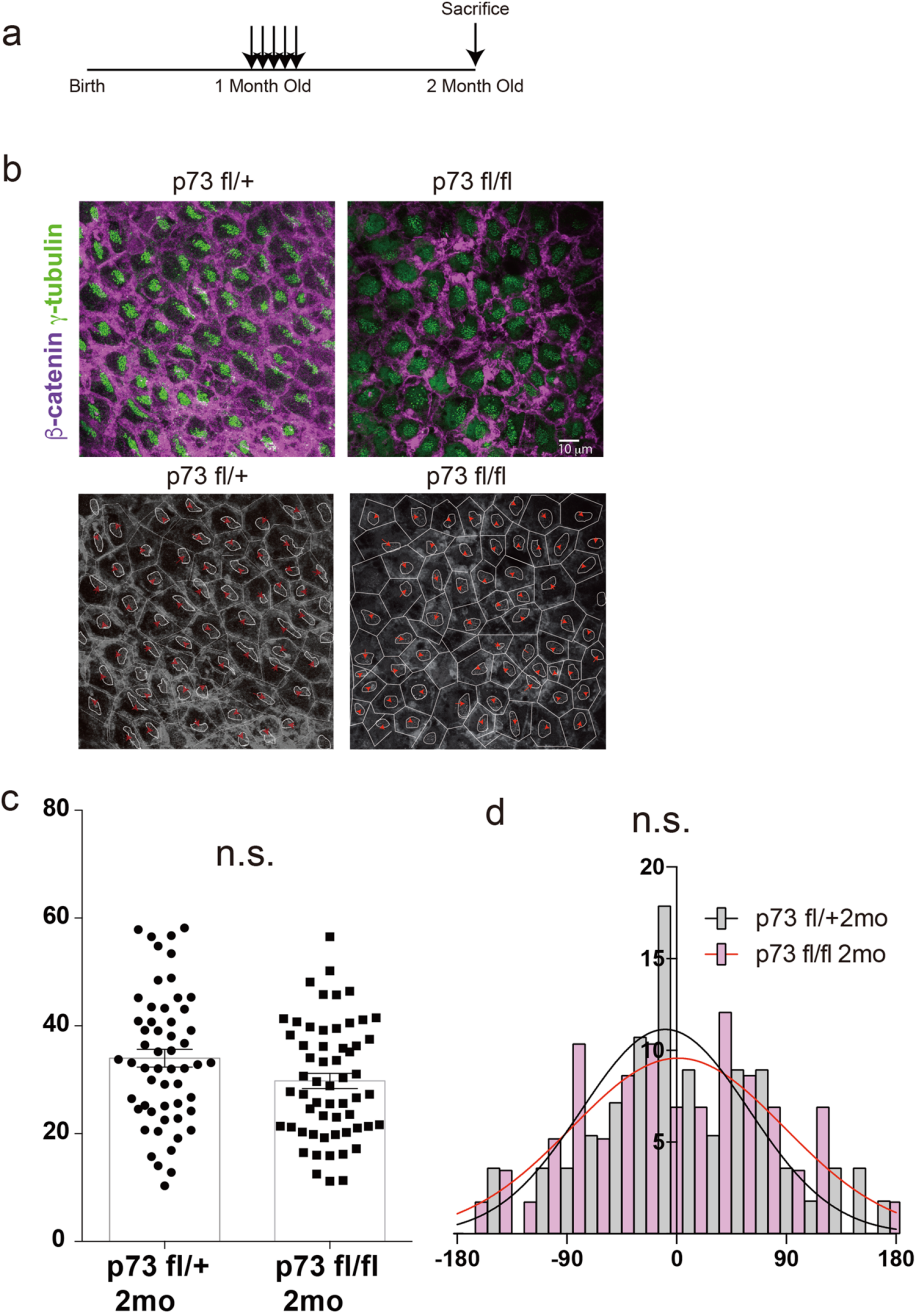
Loss of p73 in adult ependymal cells did not affect cytoskeletal architecture and translational polarity. (**a**) Schema shows experimental schedule. (**b**) Whole-mount preparations of lateral walls of lateral ventricles stained with antibodies against β-catenin (purple) and g-tubulin (green) in heterozygous (p73fl/+; p73 flox/+::FoxJ1 CreERT2 Tg/+::R26R tdTomato KI (knock-in)/+) and homozygous (p73 fl/fl; p73 flox/flox::FoxJ1 CreERT2 Tg/+::R26R tdTomato KI/+) mutant brains. Traces of intercellular junction were labeled with b-catenin, and BB were labeled with γ-tubulin of ependymal cells. Red arrows show vectors drawn from center of the apical surface to BB patch. Scale bar, 10 μm. (**c**) Quantification of BB patch displacement. (n.s. not significantly different, mean ± SEM; n = 55 p73 fl/+ mice, n = 58 p73 fl/fl mice). (**d**) Histogram of distribution of BB patch angles in heterozygous (het) (gray) and homozygous (ko) (pink) mutants (n.s. not significantly different, Watson’s U2 test, n = 55 p73 fl/+ mice, n = 58 p73 fl/fl mice).

We conditionally disrupted p73 in the ependymal cells of one month old brains, prepared whole mount lateral ventricles and performed morphological analysis of the ependymal cells and cilia at 2 months old (Fig. 4b). Ependymal cell morphology, as assessed by immunostaining for β-catenin, was not disturbed in the p73 floxed homozygous mouse (Fig. 4c). Additionally, we immunostained γ-tubulin and analyzed BB patch displacement and vector (Fig. 4c–d) but observed no significant differences between the p73 floxed heterozygous mouse and homozygous mouse. These results indicate that p73 is dispensable in the adult brain for maintenance of translational polarity and the planar polarization of BB patches on the apical surface of ependymal cells.

## Discussion

In this study, we confirmed the previously reported function of p73 and identified a new function with newly established mutant mice. An examination of the integrity of cilia and BB patch formation led us to conclude that p73 regulates embryonic translational polarity formation, ependymal cell morphology and proper aqueduct formation.

Ependymal maturation occurs via 4 steps during the perinatal period^6,31^. (1) From e14 until birth, a subpopulation of radial glia is specified to enter the ependymal cell lineage. (2) At birth, a radial glial cell begins the differentiation process. (3) At postnatal day 4, in young ependymal cells, BBs are docked; the cilia have not reached their final length and are beating randomly. (4) At postnatal day 21, cilia have reached their final length and have oriented their beating in the caudorostral direction. Because we deleted p73 from P1-P5 to target motile multiciliogenesis, p73 was lacking in the ependymal cells during step 2 and step 3. However, hydrocephalus did not occur in these mice. As the mice show aqueductal stenosis without hydrocephalus, it is strongly suggested that aqueductal stenosis does not result in complete obstruction. In our p73 KI mouse, ciliogenesis was not perturbed at a later stage, and motile multiciliogenesis and elongation of cilia were likely to be delayed but intact, even though p73 was disrupted. Thus, p73 deletion is required at step 1 for the hydrocephalic phenotype.

We and others^17^ have observed that translational polarity is abnormal in p73 knockout and homozygous mutant mice (Fig. 3c,d). Translational polarity is dramatically affected when radial glial primary cilia are ablated earlier in development^5^. In the embryo, the radial glia have a translational polarity that predicts the orientation of the mature ependymal cells^5^. A recent study also suggests that planer cell polarity (PCP) genes (*Celsr1*, *Fzd3*, and *Vangl2)* regulate cilia positioning in the apical domain (translational polarity) in radial progenitors and ependymal cells^32^. Moreover, ablation of the embryonic primary cilia by conditional deletion of *Kif3a* and *Ift88* also disrupts ependymal cilia, resulting in postnatal hydrocephalus.

Taken together, our data indicate that embryonic primary ciliogenesis may be regulated by p73 and is critical for hydrocephalus. However, the FoxJ1 knockout mouse does not show abnormal primary cilia^33^, suggesting that FoxJ1 is dispensable for primary ciliogenesis. Therefore, using a FoxJ1CreERT^2^ mouse may be inappropriate for analyzing primary ciliogenesis in the radial glia. Further examination using another Cre line to investigate primary ciliogenesis is necessary to answer this question. As shown in Supplementary Fig. 3q, p73 is expressed not only in ependymal cells but also in choroid plexus epithelial cells at postnatal ages. FoxJ1 is expressed in choroid plexus at the postnatal age^33^. Therefore, our data suggest that postnatal p73 deficiency in choroid plexus did not contribute to hydrocephalus. In addition, p73 is expressed in the cortical hem at E13.5 (our unpublished observation). These results also suggest that embryonic p73 may have an important role in the development of hydrocephalus by regulating primary cilia formation, such as in differentiation of neuroepithelium. How can p73 regulate primary cilia formation? In general, intraflagellar transport (IFT) is responsible: kinesin motors transport ciliary components from the cell body to the primary cilia (anterograde transport), and cytoplasmic dyneins transport them back from the cilia (retrograde transport)^34,35^. As shown in a recent study^36^, IFT-associated proteins and motor proteins could be the p73 targets. These results suggest that p73 may regulate primary ciliogenesis. Importantly, we observed aqueductal stenosis. Cilia dysfunction does not always lead to obstructive hydrocephalus^4,7,8^. A possible cause of aqueductal stenosis is the hyperplasia of ependymal cells in the aqueductal wall^37^. Rho family guanosine triphosphatase (GTPase) 3 (Rnd3), a member of the small Rho GTPase family, controls cell cycle progression in a Notch-dependent manner. Rnd3 inhibits Notch signaling, and loss of Rnd3 results in Notch hyperactivation and overproduction of ependymal cells^37^. However, postnatal deletion of p73 did not cause overproduction of ependymal cells (Fig. 2b). Lin *et al*.^37^ did not exclude the possibility that Rnd3 might function through Rho-kinase signaling inhibition^38^. Therefore, Rnd3 might regulate the cytoskeletal structure of ependymal cells. Another possibility is the disorganization of the ependymal cell structure caused by abnormal adherens junctions. Afadin is required for the nectin-based cell–cell adhesion site to form adherens junctions. The Afadin knockout mouse shows aqueductal stenosis and hydrocephalus due to destruction of the adherens junctions in ependymal cells^39^. Moreover, Myo IXa (Myo9a), which is an actin-dependent motor molecule with a Rho GTPase–activating (GAP) domain, is expressed in maturing ependymal cells, and its absence leads to impaired maturation of ependymal cells^40^. These reports suggest that cytoskeletal disorganization by abnormal small GTPase signaling may be related to aqueductal stenosis via regulation of the cytoskeleton of ependymal cells. We conclude that p73 is a multifunctional protein that regulates 1) embryonic translational polarity, 2) proper aqueduct formation, and 3) ependymal cell morphology. Hydrocephalus might be caused by abnormal embryonic translation polarity. Therefore, p73 disruption in the embryonic period models human congenital hydrocephalus.

In addition to ependymal cell functions, cell death or hippocampal phenotype is also observed in p73 knockout mice^13–16,18,41^. Cell-specific conditional ablation of p73 will facilitate our understanding of the cellular and molecular mechanisms of the p73 regulatory system, not only in the brain but also in other tissues.

## Materials and Methods

### Transgenic animals

The ES cells used in this study were generated by trans-NIH KOMP and obtained from the KOMP Repository (www.komp.org). NIH grants to Velocigene at Regeneron, Inc. (U01HG004085) and the CSD Consortium (U01HG004080) funded the generation of gene-targeted ES cells for 8,500 genes in the KOMP Program, which were archived and distributed by the KOMP Repository at UC Davis and CHORI (U42RR024244). The ES cells obtained from KOMP (Clone number: EPD0851_4_D04 and E03; the targeting strategy is described in Supplementary Fig. 1a) were microinjected at the NPO Biotechnology Research and Development in Osaka University into ICR mouse blastocysts to establish germ-line transmission. The resulting chimeric offspring were bred with C57BL6/J mice to obtain germline transmission of the mutated trp73 allele and p73 KI mice. As illustrated in Supplementary Fig. 1b, to obtain a p73 floxed mouse, a p73 KI mouse was crossed with a FLPe Tg mouse obtained from NPO Biotechnology Research and Development in Osaka University with the permission of Dr. A. Francis Stewart. The p73 floxed mouse was crossed with a FoxJ1CreERT^2^::R26R-tdTomato double transgenic mouse obtained from Dr. Jonas Frisen’s lab at the Karolinska Institute to obtain p73 floxed:: FoxJ1CreERT^2^:: R26R-tdTomato triple transgenic mice (Supplementary Fig. 1b).

The mice were bred and maintained at the experimental animal facility of Osaka University Graduate School of Medicine. We euthanized the mice by injecting an overdose of a mixture of Vetorphale (0.5 mg/mL, Meiji), Dormicum (0.4 mg/mL, Roche) and Domitor (0.03 mg/mL, Orion Pharma) by peritoneal injection. The institutional committee of Osaka University approved this study, and all experiments were performed in accordance with the Guide for the Care and Use of Laboratory Animals of the Osaka University Medical School.

### Tamoxifen injection

Tamoxifen (Sigma-Aldrich) was dissolved in corn oil at a concentration of 20 mg/ml by shaking overnight at 37 °C. For adult mice, 100 µL tamoxifen/corn oil was administered once a day for 5 days^28^. For postnatal mice, 100 µL tamoxifen/corn oil was administered to lactating mothers once a day for 5 days^29^.

### RT-PCR

Tissues were homogenized in TRIzol reagent (Thermo Fisher Scientific). Isolated total RNA was purified with an RNeasy Micro kit (QIAGEN). The complementary DNA (cDNA) was synthesized with a High Capacity cDNA Reverse Transcription kit (Applied Biosystems). A reaction without template was used as a negative control.

### Real-time PCR analysis

TaqMan real-time PCR analysis was conducted using TaqMan Universal Master Mix II (Thermo Fisher Scientific) with specific probe mixtures for each gene (*p73*: Mm01263882_m1 (which targets the boundary of exon 3’ and 4) and glyceraldehyde-3-phosphate dehydrogenase (*Gapdh*): Mm99999915_g1). The expression of each gene was normalized to *Gapdh*. The reaction and subsequent quantification were conducted using a QuantStudio 7 Flex Real-Time PCR System (Applied Biosystems).

### Nissl staining

Mice were euthanized and transcardially perfused with PBS followed by 4% paraformaldehyde. After post fixation at 4 °C overnight, brains were cryoprotected and sectioned at 40 μm for adults and 20 μm for e18.5 embryos. After placing floating sections on the slides and drying the slides, Nissl staining was performed using a previously described method^13^. Images were acquired with a BZ-9000 fluorescence microscope (Keyence).

### X-gal staining

Brains were prepared as described above. After postfixation for 1 hour at 4 °C, brains were washed with PBS, cryoprotected overnight and sectioned at 40 μm for adults and 20 μm for pups at postnatal day 5. Sections were immersed in a detergent rinse (0.1 M PB, 2 mM MgCl_2_, 0.01% sodium deoxycholate, 0.02% NP-40) for 20 min and stained with X-gal staining buffer (0.1 M PB, 2 mM MgCl_2_, 0.01% sodium deoxycholate, 0.02% NP-40, 5 mM K_3_Fe(CN)_6_, 5 mM K_4_Fe(CN)_6_, 20 mM Tris) at 37 °C for 48 hours. Images were acquired with a BZ-9000 fluorescence microscope (Keyence).

### Whole mount preparation of lateral ventricle wall and immunostaining

Whole mounts of the lateral ventricle walls were prepared as previously described^5,7,30,42^. The exposed walls were fixed in 4% PFA at 4 °C overnight. After blocking, whole mounts were incubated with primary antibodies in the blocking solution overnight at 4 °C, washed, and incubated with secondary antibodies in the blocking solution overnight at 4 °C.

### Antibodies

The primary antibodies were mouse anti-β-catenin (1:1000, BD Transduction Laboratories 610153), rabbit anti-β-catenin (1:1000, Sigma C2206), mouse anti-acetylated tubulin (1:1000, Sigma T6793), and rabbit anti-γ-tubulin (1:1000, Sigma T5192). Secondary antibodies were conjugated to Alexa Fluor dyes (goat polyclonal, 1:500, Invitrogen).

### BB analysis

Confocal images were taken on an FV-1200 (Olympus). To analyze BB patch angle and BB rotational orientation, vectors were drawn using ImageJ (National Institutes of Health) software as previously described^5^. The angles of the vectors were normalized such that the average of the angle of the vectors was 180° in each E cell. The percentage of vectors was plotted on a histogram in 15° bins. BB displacement was quantified using ImageJ software (National Institutes of Health). To normalize the magnitude of the vector for the size and shape of the apical surface in each E cell, we divided it by the length of a line drawn from the center of the apical surface to the edge of the apical surface, passing through the center of the BB patch.

## Electronic supplementary material

Supplementary Information
