# Supplementary material for: Loss of p73 in ependymal cells during the perinatal period leads to aqueductal stenosis
Source: Sci Rep. 2017 Sep 20;7:12007. doi: 10.1038/s41598-017-12105-z (PMC5607290; doi:10.1038/s41598-017-12105-z)
Supplement: Supplementary file 1 — Supplementary Information [file 41598_2017_12105_MOESM1_ESM.pdf]

Supplementary Information

Loss of p73 in ependymal cells during the perinatal period leads to aqueductal stenosis

Masashi Fujitani[1, 2, 3*], Ryohei Sato[1], Toshihide Yamashita[1*]

[1]Department of Molecular Neuroscience, Graduate School of Medicine, Osaka University, 2-2 Yamadaoka, Suita, Osaka 565-0871, Japan

[2]Molecular Research Center for Children's Mental Development, United Graduate School of Child Development, Osaka University, 2-2 Yamadaoka, Suita, Osaka 565-0872, Japan

[3]Department of Anatomy and Neuroscience, Hyogo College of Medicine, 1-1 Mukogawa-cho, Nishinomiya, Hyogo 663-8501, Japan

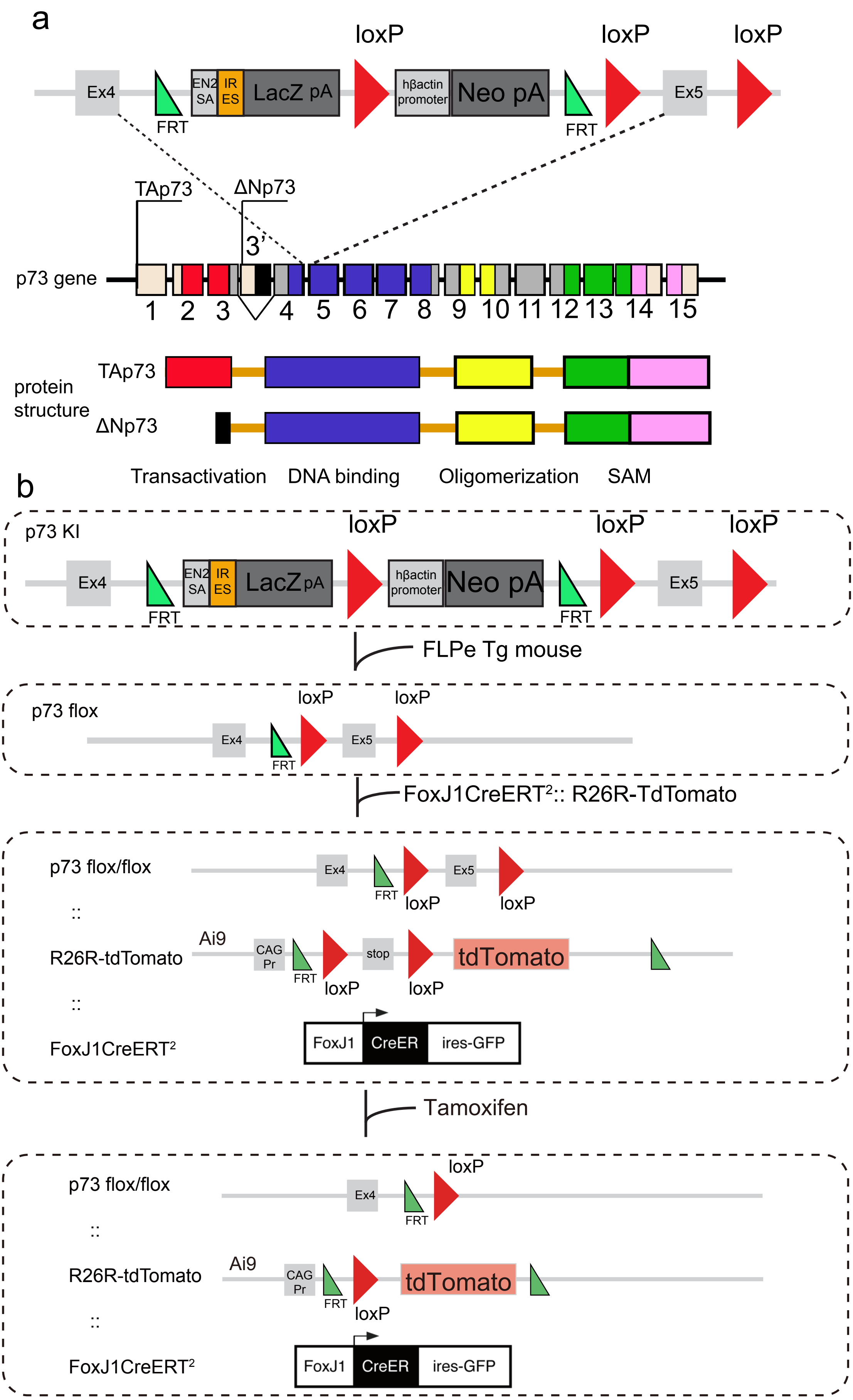

Supplementary Figure 1: Target and breeding strategy for the p73 mutant mice in this study. (a) An ENGRAILED 2 splice acceptor (EN2SA), LacZ and neomycin expression cassette were inserted into intron 4 of the mouse p73 gene to obtain a p73 knock-in mouse (p73 KI). Protein structures of both isoforms of p73 were represented (TAp73: full length, ΔNp73: N-terminal truncated isoform).(b) To obtain a conditional knockout mouse, we first crossed our knock-in mouse with the FLPe transgenic mouse (FLPe Tg mouse) to excise the expression cassette. A p73 floxed mouse was generated and crossed with a FoxJ1CreERT^2::R26R-tdTomato mouse obtained from the Jonas Frisen lab[25]. To activate CreERT^2 in the ependymal cells, tamoxifen was administered to the mouse.

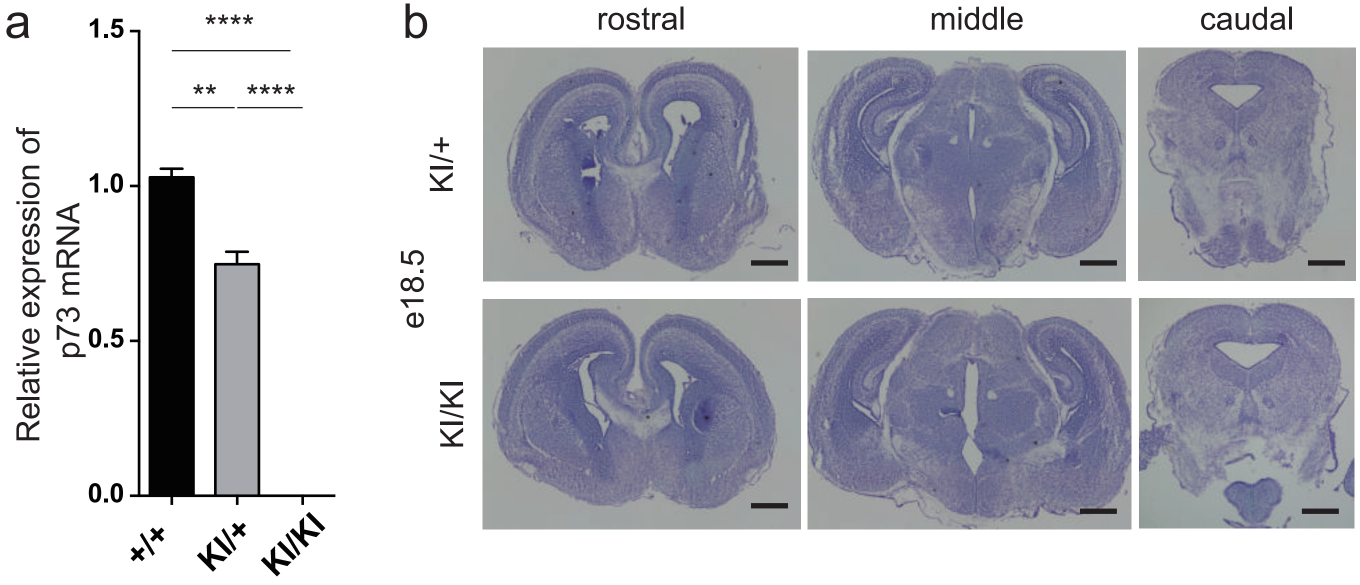

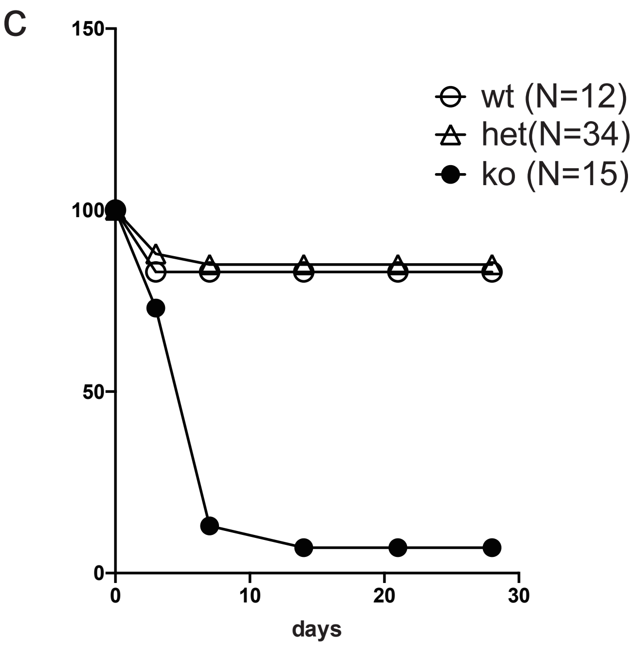

Sypplementary Figure 2. Confirmation of a p73 knock-in (p73 KI) mouse phenotype.
(a) Expression of p73 mRNA in postnatal cortices from wild type (+/+), heterozygous (KI/+) and homozygous (KI/KI) mutants. **$p<0.01$ **** $p<0.0001$ (n=3, One-way ANOVA, Tukey-Kramer test).
(b) Nissl staining of coronal sections of the rostral, middle, and caudal parts of embryonic day (e) 18.5 brains of both heterozygous (p73 KI/+) and homozygous (p73KI/KI) mutants. Scale bars, 500 μm.

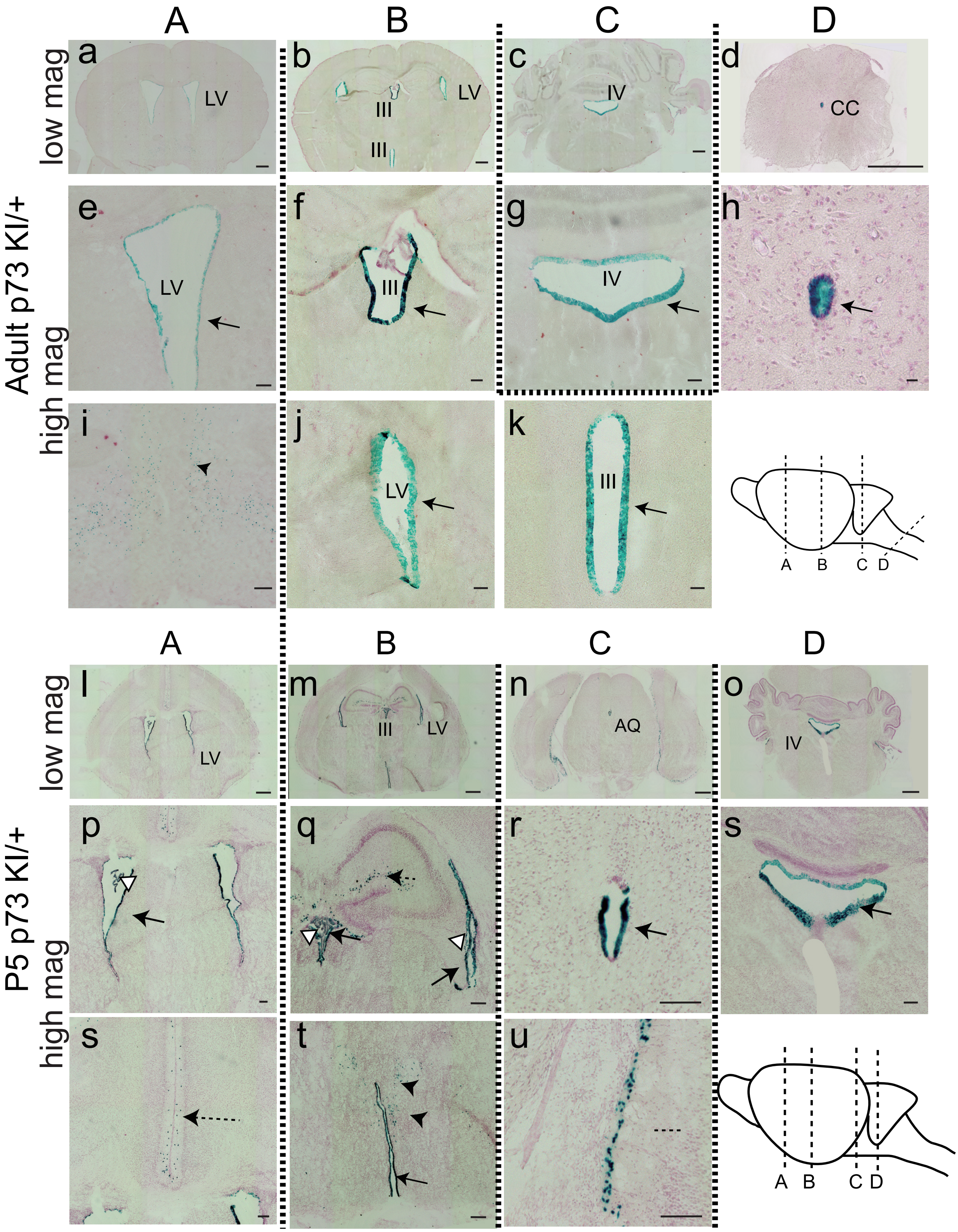

Sypplementary Figure 3. Figure 2. p73 expression in adult and postnatal brains (a-k) X-gal staining of coronal sections of the rostral (A: a, e and i), middle (B: b, f, j and k), caudal (C: c and g) parts of the adult brain and spinal cord (D: d and h) of heterozygous (p73 KI/+) mutants. Black arrows indicate the ependymal cell layer, and the arrowhead indicates hypothalamus cells. LV, lateral ventricles; III, third ventricle; IV, fourth ventricle; CC, central canal; AQ, aqueduct. Scale bars, a-d: 500 µm, e-k: 100 µm. (l-u) X-gal staining of coronal sections of the rostral (A: l, p and s), middle (B: m, q and t), caudal (C: n, r and u) parts of the forebrain and hindbrain (D: o and s) of postnatal day 5 (P5) brain. Black arrows indicate the ependymal cell layer; the white arrowhead indicates the choroid plexus; dotted arrows indicate Cajal-Retzius cells; and the black arrowhead indicates hypothalamus cells. Scale bars, l-o: 500 µm, p-u: 100 µm.

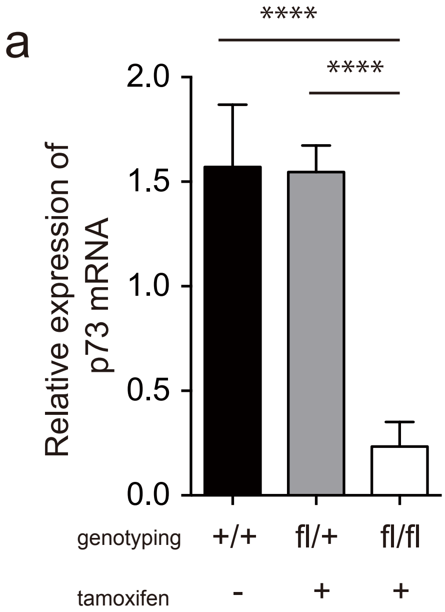

Supplementary Figure 4: (a) Expression of p73 mRNA in ependymal and subventricular zone from non-tamoxifen injected wild type (+/+), tamoxifen-injected heterozygous (fl/+) and homozygous (fl/fl) mutants at one month old. ****$p<0.0001$ (n=3, One-way ANOVA, Tukey-Kramer test).
